# Detection of Chronic Wasting Disease Prions in Prairie Soils from Endemic Regions

**DOI:** 10.1021/acs.est.4c04633

**Published:** 2024-06-12

**Authors:** Alsu Kuznetsova, Anthony Ness, Erin Moffatt, Trent Bollinger, Debbie McKenzie, Iga Stasiak, Charlie S. Bahnson, Judd M. Aiken

**Affiliations:** † Department of Renewable Resources, University of Alberta, Edmonton T6G 2G7, Canada; ‡ Centre for Prions and Protein Folding Diseases, University of Alberta, Edmonton T6G 2M8, Canada; § Canadian Wildlife Health Cooperative Western Northern, University of Saskatchewan, Saskatoon S7N 5B4, Canada; ∥ Ministry of Environment, Government of Saskatchewan, Saskatoon S7N 3R3, Canada; ⊥ North Dakota Game and Fish Department, Bismarck, North Dakota 58501-5095, United States

**Keywords:** chronic wasting disease, prairie soils, prions, prion diseases, deer, cervids

## Abstract

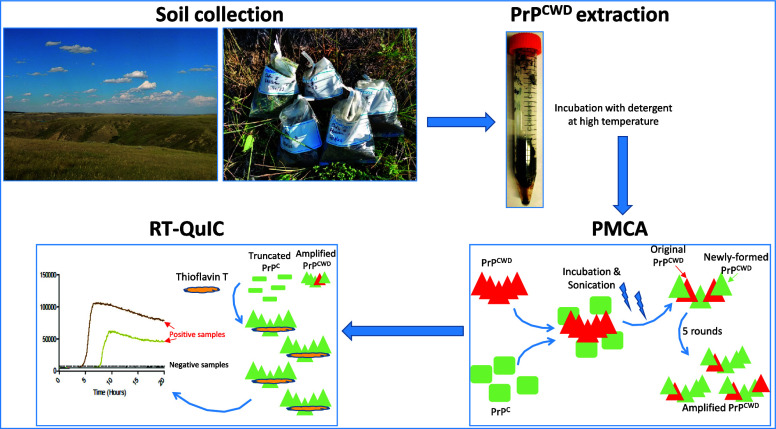

Chronic wasting disease (CWD) is a contagious prion disease that affects cervids in North America, Northern Europe, and South Korea. CWD is spread through direct and indirect horizontal transmission, with both clinical and preclinical animals shedding CWD prions in saliva, urine, and feces. CWD particles can persist in the environment for years, and soils may pose a risk for transmission to susceptible animals. Our study presents a sensitive method for detecting prions in the environmental samples of prairie soils. Soils were collected from CWD-endemic regions with high (Saskatchewan, Canada) and low (North Dakota, USA) CWD prevalence. Heat extraction with SDS-buffer, a serial protein misfolding cyclic amplification assay coupled with a real-time quaking-induced conversion assay was used to detect the presence of CWD prions in soils. In the prairie area of South Saskatchewan where the CWD prevalence rate in male mule deer is greater than 70%, 75% of the soil samples tested were positive, while in the low-prevalence prairie region of North Dakota (11% prevalence in male mule deer), none of the soils contained prion seeding activity. Soil-bound CWD prion detection has the potential to improve our understanding of the environmental spread of CWD, benefiting both surveillance and mitigation approaches.

## Abstract 2

Detection of Chronic Wasting Disease in soil samples is a critical first step in identifying contaminated regions for development and deployment of environmental mitigation strategies.

## Introduction

Chronic wasting disease (CWD) is a contagious prion disease affecting free-ranging and farmed cervids in North America, Europe, and South Korea. CWD has been detected in 32 states in the United States, 5 Canadian provinces (https://www.usgs.gov/media/images/distribution-chronic-wasting-disease-north-america-0) and in 3 Scandinavian countries.^1,2^ In CWD-endemic areas, the disease prevalence in free-ranging deer herds can exceed 50–75%, negatively affecting cervid populations.^3,4^

Direct and indirect (environmental) horizontal transmission is the principal modes of CWD spread.^5^ CWD prions have been detected in saliva, urine, and feces of both clinical and nonclinical animals.^6−8^ One remarkable property of CWD prions (PrP^CWD^) is their persistence in external environments and their ability to remain infectious for years.^9,10^ Repopulation of cervids to CWD-contaminated areas can facilitate CWD transmission.^11^ Soils are a natural environmental reservoir of shed PrP^CWD^ and contribute to indirect transmission of CWD.^12−14^ Prion detection in soils is challenging as prions can bind avidly to soils, and recovery of soil-bound PrP^CWD^ becomes more difficult with time^15,16^; however, PrP^CWD^ detection in soils is necessary for identifying CWD-contaminated lands.^8,17,18^ Environmental conditions, such as multiple freeze–thaw or dry–wet cycles, exposure to indigenous microbes and enzymes, extreme soil pH, and adsorption to soil particles, may affect of PrP^CWD^ detectability and persistence over time.^17,19^ At the same time, the binding to soil particles increases biological uptake and retention of the prions and can enhance disease transmission.^17,20^ The extent to which environmental contamination contributes to disease transmission in wild cervid populations remains unknown. Given that prions are released into the environment and environmental transmission plays a role in CWD infection, it is critical to be able to detect environmental PrP^CWD^. Moreover, by monitoring relative PrP^CWD^ levels in soil, we would be able to assess CWD persistence in the environment and effectiveness of control programs.

The CWD minimum infectious dose approximates to 100–300 ng of CWD-positive brain,^21^ and that amount could be a threshold for the detection in the environmental samples. There are sensitive methods and techniques that allow for detection of low amounts of PrP^CWD^ in different materials: real-time quaking-induced conversion (RT-QuIC) for saliva, urine, and spiked environmental surfaces^22^; and serial protein misfolding amplification (PMCA) for feces,^19^ muscle tissue, spiked soils,^23−26^ invertebrates,^27^ or vegetation grown in the laboratory.^28^ These techniques result in an increase of the detection limit of CWD prions by 4 orders of magnitude. However, the complexity and variability of environmental samples (e.g., soils) can interfere or inhibit these *in vitro* amplification assays more than in biological samples.^29^ Thus, the major challenge is to extract PrP^CWD^ bound to soils that retain the assay seeding activity.

The aim of this study was to develop and apply a sensitive method to detect PrP^CWD^ in soils collected from CWD-endemic regions to contrast soils from an area in Saskatchewan (Canada) where CWD prevalence in free-ranging cervid populations is high to a region where prevalence is low (North Dakota,USA). Positive detection of CWD prions in soils collected in 2 consecutive years from sites with recent deer activity in an area with high CWD prevalence was a key finding.

## Material and Methods

### CWD-Endemic Regions: Study Sites and Soil Collection

Soil samples were collected from CWD-endemic regions with high (South Saskatchewan (SK), Canada) and low (North Dakota (ND), USA) disease prevalence in wildlife. White-tailed deer and mule deer cohabit these areas. The South Saskatchewan River Valley (SSRV) region has a CWD prevalence (2020–2022) in mule deer of over 70% in males (95% confidence interval, 70–84%) and over 30% in females (95% confidence interval, 32–53%) (https://www.saskatchewan.ca/residents/environment-public-health-and-safety/wildlife-issues/fish-and-wildlife-diseases/chronic-wasting-disease/cwd-map). In contrast, CWD prevalence rates in ND are considerably lower; CWD was first detected in a mule deer buck in 2018. Twenty-one additional detections were found through 2022, consisting of 19 mule deer bucks, one mule deer doe, and one white-tailed buck. Within a 9961 km^2^ area that includes Divide County and portions of Burke, Mountrail, and Williams Counties, the reported prevalence of CWD (2020–2022) among adult, hunter-harvested mule deer was 11% (95% confidence interval, 7–18%) in males and 0.5% in females (95% confidence interval, 0.1–3%).

The South SK landscapes are mixed grasslands prairie and rolling plains blanketed with a layer of coarse glacial until or fine-textured ancient glacial lake deposits and are carved by coulees formed by glacial meltwater. Northwest ND consists of mixed-grass prairie, characterized by rolling hills with shallow wetland basins.^30^ The climates of both prairie regions are semiarid with short warm summers and long cool winters. The average annual daily temperature is 4 °C with a mean annual precipitation amount of 352 mm. Most of this precipitation falls between the months of May and September. Soil types in this ecoregion are of the chernozemic order, more specifically, Brown Chernozems.^31^ This ecoregion has relatively little addition of a coarse organic matter that decomposes faster into the soils compared to the soils to the north. This is due to the relatively warmer temperatures. Soils are thinner on the upper slopes and thicker on the lower portion of the slopes. For both south SK and northwest ND, land use primarily consists of farming, ranching, and energy development. The sampling area in SK is located on the right bank of the South Saskatchewan River and occupies approximately 200 km^2^; the sampling area in ND is located in northwest corner of the Divide County and occupies approximately 100 km^2^.

Soil samples were collected from both public and private lands. Landowners and farmers were consulted to identify areas where deer were gathered. Soil collection sites were distinguished as with or without recent deer activity using remote cameras, visual monitoring, and GPS collars. As a negative control, soils (e.g., Soil 1 in Table S2) with similar properties were collected in the central part of Alberta (Canada) from a harvested field in 2010 (when CWD was not endemic in this area). Soil samples from the endemic regions were collected from the surface (0–5 cm) on a grid, 5 subsamples 1 m apart from each site. Samples were collected from a combined total of 25 sites in SK and 13 sites in ND at the end of vegetation seasons in 2021 and 2022 (Table S1). Locations of the sites were spread out across farmlands including fields and yards, as well as natural surroundings, predominantly within valleys of rivers and creeks (coulees). All soils collected for CWD analyses have similar properties to the soil used as a negative control—loam clay texture, slightly alkaline pH varying from 6.3 to 7.9, low total organic carbon (<3.9%), and blocky structure (Table S2).

### Spiked Soil—CWD Prion Detection

For the optimization of soil prion detection, spiked soils were used. Mineral horizons of prairie (Chernozem, Soil 1 in the Table S2) and boreal (Luvisol, Soil 2 in the Table S2) soils collected in 2010 from a region free of CWD were used for the method optimization. The soils were described previously^26^ and have similar properties as those collect in CWD-endemic regions. CWD prion homogenates were prepared from the CWD prion-infected brain tissue of transgenic mice expressing elk PrP (TgElk) or from hunter-harvested CWD-infected mule deer. Uninfected brain tissue from tgElk mice served as negative controls. Brains were homogenized (10% (w/v)) in water. Air-dried soils (100 mg) were spiked with a specific amount of 1% tgElk-CWD BH (10, 5, 1, or 0.5 μL) and incubated at 4 °C in the dark. After incubation with soils, PrP was extracted with SB or other tested solutions at 80 °C for 10 min and 10 μL of supernatant was used as a seed for PMCA or PrPCWD was directly detected by immunoblotting.

### Immunoblotting

Samples were resolved on 15-well 12% NuPAGE bis-Tris gels (Invitrogen, USA), transferred to polyvinylidene difluoride membranes, and probed with an anti-PrP antibody (Bar 224 at 1:20,000). Blots were developed using the AttoPhos AP Fluorescent Substrate System (1:10,000; Promega Corporation, USA). Fluorescent imaging of the membranes was performed using an ImageQuant LAS 4000 (GE Life Sciences, USA) system.

### Prion Extraction from Soils

Soil samples were mixed with SDS-containing buffer hereafter named as SB (300 mM tris base, 50% (v/v) glycerol, 10% (w/v) sodium dodecyl sulfate, 25% (v/v) β-mercaptoethanol) in a 1:1 ratio. Samples were vigorously shaken to ensure homogeneous mixing of soil with the extraction buffer and heated for 10 min at 80 °C. The samples were allowed to cool; a brief centrifugation was then performed to settle solid particles (1000 rpm for 1 min). The sample supernatant (10 μL) was used to seed tgElk PMCA substrate.

### Protein Misfolding Cyclic Amplification (PMCA) Assay

PMCA was employed to amplify PrP^CWD^ extracted from soils. Perfused uninfected tgElk brain homogenate (BH) was used as a substrate. An amplification control of 10% CWD-infected BH samples were serially diluted 10-fold in 10% noninfective BH. Each PMCA reaction included two 3/32″ PTFE beads (McMaster-Carr, USA) to increase the efficiency of prion amplification.^32^ Subsequently, 90 μL of each 10-fold dilution series and a negative control (10% uninfected BH) were placed in a QSonica Q700 sonicator (Misonix Inc., Farmingdale, USA). Samples were incubated at 37 °C and subjected to a round of amplification with 96 cycles of 30 s sonication followed by 15 min incubation. Identical samples were incubated at 37 °C for the same period without sonication as for a non-PMCA control. Subsequent rounds of PMCA were performed under the same conditions using a 1:10 dilution of amplified materials from the previous round in 10% uninfected tgElk BH substrate. After 4–5 rounds of PMCA, the amplification products and non-PMCA controls were digested using 75 μg/mL proteinase K (PK) for 1 h at 37 °C with agitation to detect misfolded residual PrP (PrP^res^). Digestion was terminated by the addition of 5× Laemmli sample buffer (150 mM Tris-HCl (pH 6.8), 0.5% bromophenol blue, 25% (v/v) glycerol, 5% (w/v) SDS, 12.5% (v/v) β-mercaptoethanol) and boiling at 100 °C for 10 min. Samples were analyzed for evidence of PrP^CWD^ seeding by immunoblotting.

### Real-Time Quaking Induced Conversion (RT-QuIC) Assay

RT-QuIC reactions used a recombinant truncated Syrian hamster PrP substrate (amino acids 90–231) (MNPRO, USA). RT-QuIC reactions were set up using 2 μL of sample in 98 μL of assay buffer in Nunc MicroWell 96-Well Optical-Bottom Plates (Thermo Scientific, USA). Final RT-QuIC reactions contained 0.1 mg/mL hamster PrP substrate, 11.9 mM sodium phosphates, 307 mM NaCl, 2.7 mM KCl, 1 mM EDTA, 10 μL of thioflavin T (ThT), and 0.002% sodium dodecyl sulfate (w/v), at pH 7.4. Fifth-round PMCA products were diluted 100-fold, and then 2 μL was used to seed RT-QuIC reactions in sextuplicate. Uninfected white-tailed deer and CWD-infected mule deer BH (0.1% w/v) were used as negative and positive controls.

Plates were sealed with a Microseal “B” PCR Plate Sealing Film (Bio-Rad, USA) and placed in a FLUOstar Omega fluorescence plate reader (BMG LABTECH GmbH, Germany) preheated to 42 °C. The RT-QuIC assay was run for a total of 20 h with cycles of 5 min of double orbital shaking (700 rpm) and resting while scanning for thioflavin ThT signal (450 nm excitation, 480 nm emission). The positive sample threshold was calculated using the average ThT fluorescence signals of the negative brain homogenate control +10 standard deviations. Samples with at least 3 of 6 replicates above the threshold are considered positive. Values were plotted as the average of sextuplicate reactions using GraphPad Prism software (GraphPad Software, USA).

## Results and Discussion

### Detection of PrP^CWD^ in Spiked Soil Samples

A number of extraction solutions (SB, 10% SDS, 5% SDS, 2% urea, and deionized water) were compared for their ability to recover PrP^CWD^ seeding activity from prion contaminated soils (Figure S1). After 24 h incubation of CWD BH with minerals and soils, we treated samples with the extraction solutions. Three minerals (quartz, illite, and montmorillonite) and 2 soils (Luvisol from boreal region and Chernozem from a prairie region) were tested. Overall, the optimal PrP^CWD^ recovery occurred after extraction with SB and SDS solutions (Figure S1). Quartz has negligible binding capacity to prions; thus, PrP^CWD^ was effectively extracted from quartz and detected by immunoblot. For both Illite and montmorillonite, SB extracted more PrP^CWD^ compared to water, approximately 28 and 10%, respectively (Figure S1B). Comparing boreal and prairie soils, the overall PrP^CWD^ recovery was better from the boreal soil. For boreal soil, Luvisol, the strongest signal was for SB (100%), then for SDS solutions (5–10%); for prairie Chernozem, the recovery signal was almost equal for SB and SDS solutions (8–10%, Figure S1). Based on these findings, we opted to employ the SDS-containing buffer (hereafter SB), containing 10% SDS, for the extraction of PrP^CWD^ from soil samples.

**Figure 1:**
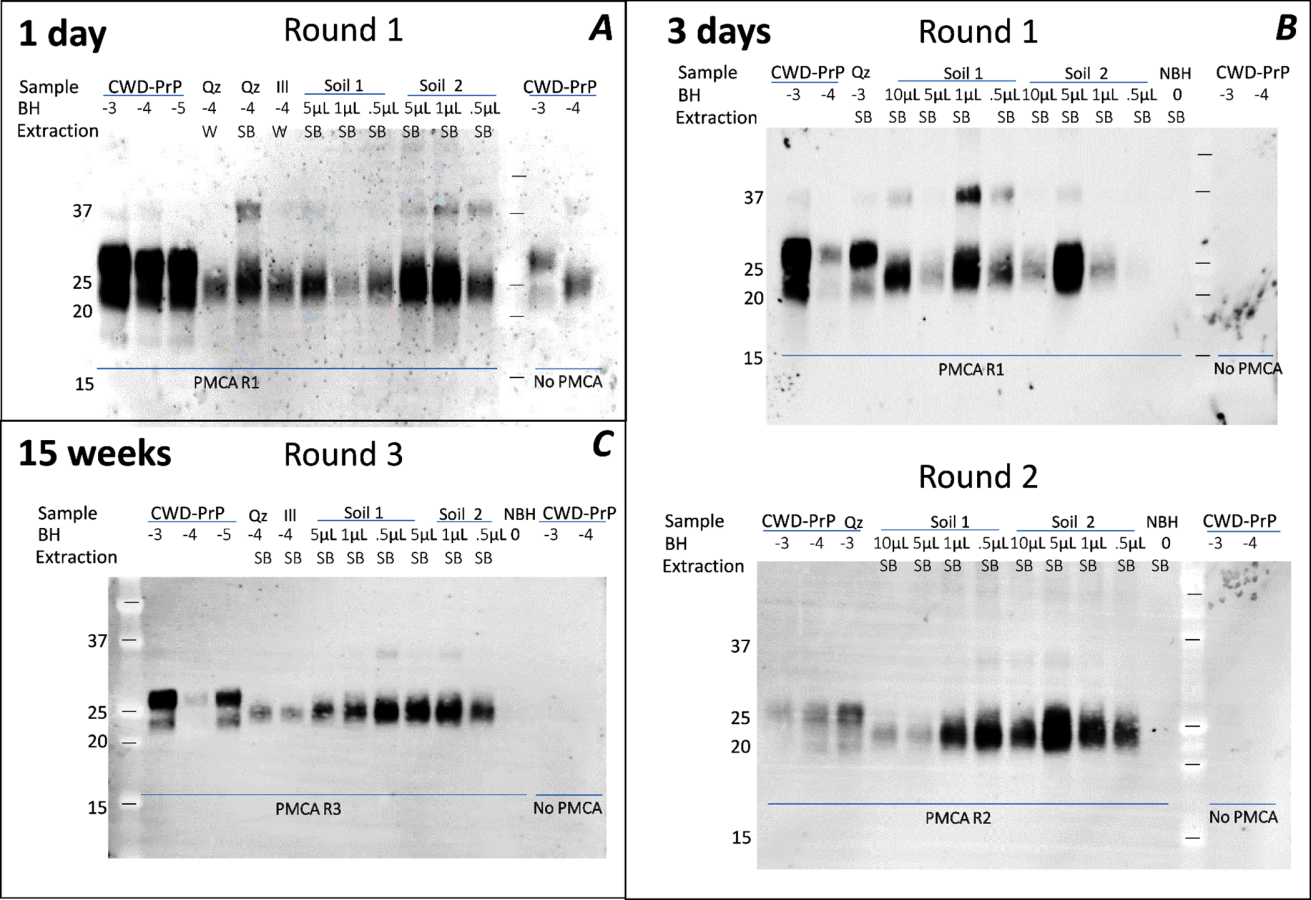
PrP^CWD^ detection in spiked soils using PMCA. Soils (100 mg) were spiked with 1% tgElk-CWD BH (10, 5, 1, or 0.5 μL) and incubated at 4 °C in the dark for 1 day (panel A), 3 days (panel B), and 15 weeks (panel C). PrP was extracted from soils with SB at 80 °C for 10 min, and 10 μL of supernatant was used as a seed for PMCA. Quartz (Qz) and illite (Ill) samples were also subjected to water extraction (W), and these supernatants were used as a seed for PMCA (Panel A). CWD BH in dilution 10^–3^–10^–5^ and uninfected BH (NBH, 10^–3^) were used as PMCA amplification controls. PMCA products were PK-digested (50 μg/μL) and immunoblotted with mAb Bar224.

Initially, we tested this approach with soils artificially spiked with PrP^CWD^. Spiked soils were extracted with SB, and the signal was amplified by PMCA (Figure 1). Boreal soils (sandy and loamy) or minerals (100 mg) were spiked with 1% tgElk-CWD BH (5, 1, or 0.5 μL) and incubated at 4 °C in the dark for 1 day (Figure 1A), 3 days (Figure 1B), and 15 weeks (Figure 1C). PrP was extracted from soils with SB, and 10 μL of supernatant was used as a seed for PMCA. Uninfected BH (NBH) spiked to one of the soils was used as a control. After 1 day of incubation, subsequent extraction with SB and 1 round of PMCA, we detected PrP^CWD^ in all CWD spiked samples. Spiking these low amounts (e.g., 0.5 μL of BH to 100 mg of soil) is not detectable by immunoblot without PMCA.^15^ After 3 days of incubation, binding became more avid and it was more difficult to extract entire amount of PrP. The first round of PMCA showed signals for Soil 1 spiked samples, but for Soil 2, only 5 μL amplified well and 0.5 μL was not detectable. However, the second round of PMCA showed strong signals for both soils. Even after 15 weeks of incubation, we detected 0.5 μL of PrP^CWD^ spiked into both soils. It is noteworthy that the use of SB extraction did not appear to inhibit PrP^CWD^ seeding activity by PMCA (Figure 1, Figure S2). Previous bioassays have demonstrated that the prion infectivity attack rate remains 100% after heated SDS treatment similar to the soil extraction methods employed.^33^

**Figure 2:**
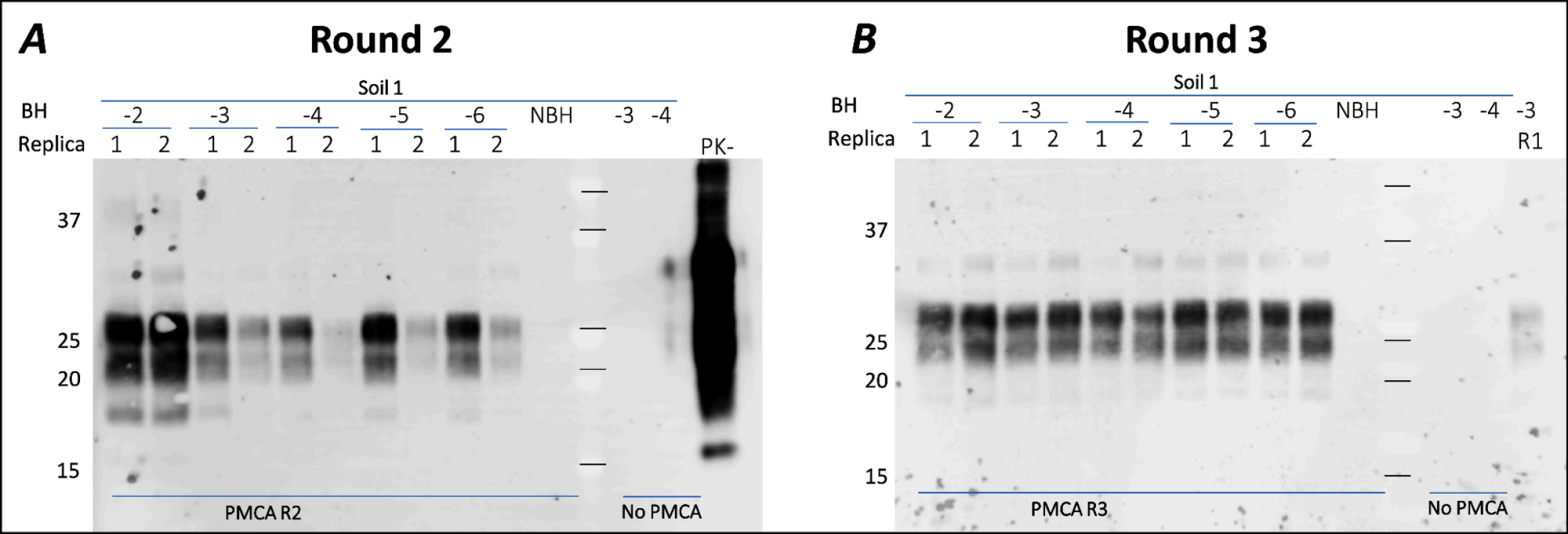
sPMCA detection limit—PrP^CWD^ at least at the 10^–6^ level of dilution is detectable after 3 rounds of PMCA. Soil (100 mg) was spiked with 1% tgElk-CWD BH to make final concentration of PrP^CWD^ at the 10^–2^–10^–6^ level and incubated at 4 °C at the dark for 1 day. PrP was extracted from soil with SB, and 10 μL of supernatant was used as a seed for sPMCA. Samples with dilution 10^–2^–10^–3^ were incubated without sonication as a “No PMCA” control. Uninfected BH (10^–3^) was used as a seed for the negative control (NBH). PMCA products after round 2 (panel A) and round 3 (panel B) were PK-digested (50μg/μL) and immunoblotted with mAb Bar224.

To quantify the detection limit, we performed a serial PMCA (sPMCA) reaction with sandy boreal soil (Luvisol). The soil (100 mg) was spiked with dilutions of a 1% tgElk-CWD BH to make final PrP^CWD^ concentration at 10^–2^–10^–6^. The spiked samples were incubated 4 °C in the dark for 1 day, and PrP was extracted with SB; 10 μL of supernatant was used as a seed for sPMCA. Samples with dilutions of 10^–2^–10^–3^ were incubated without sonication as “no PMCA” controls. Uninfected BH spiked and extracted from the same soil was used as a seed for the negative control (NBH). After 2 rounds of PMCA, at least 1 replicate of spiked samples showed detectable signal for the whole range of samples, including 0.001×, ∼equal to 10^–6^ μg of PrP^CWD^ bound to the soil (Figure 2). After 3 rounds of PMCA, both replicates for each dilution showed strong signals. Thus, we can identify the sPMCA PrP^CWD^ detection limit at 10^–6^ or lower after 3 rounds of PMCA. A dilution of 1 × 10^–6^ was selected as a target reference point for CWD detection in spiked soils. The complexity of field soil samples is expected to result in the detection limit of the method depending on soil properties, sampling locations, and the time elapsed since the soil was contaminated.

### Detection of PrP^CWD^ in Environmental Soil Samples

**Figure 3:**
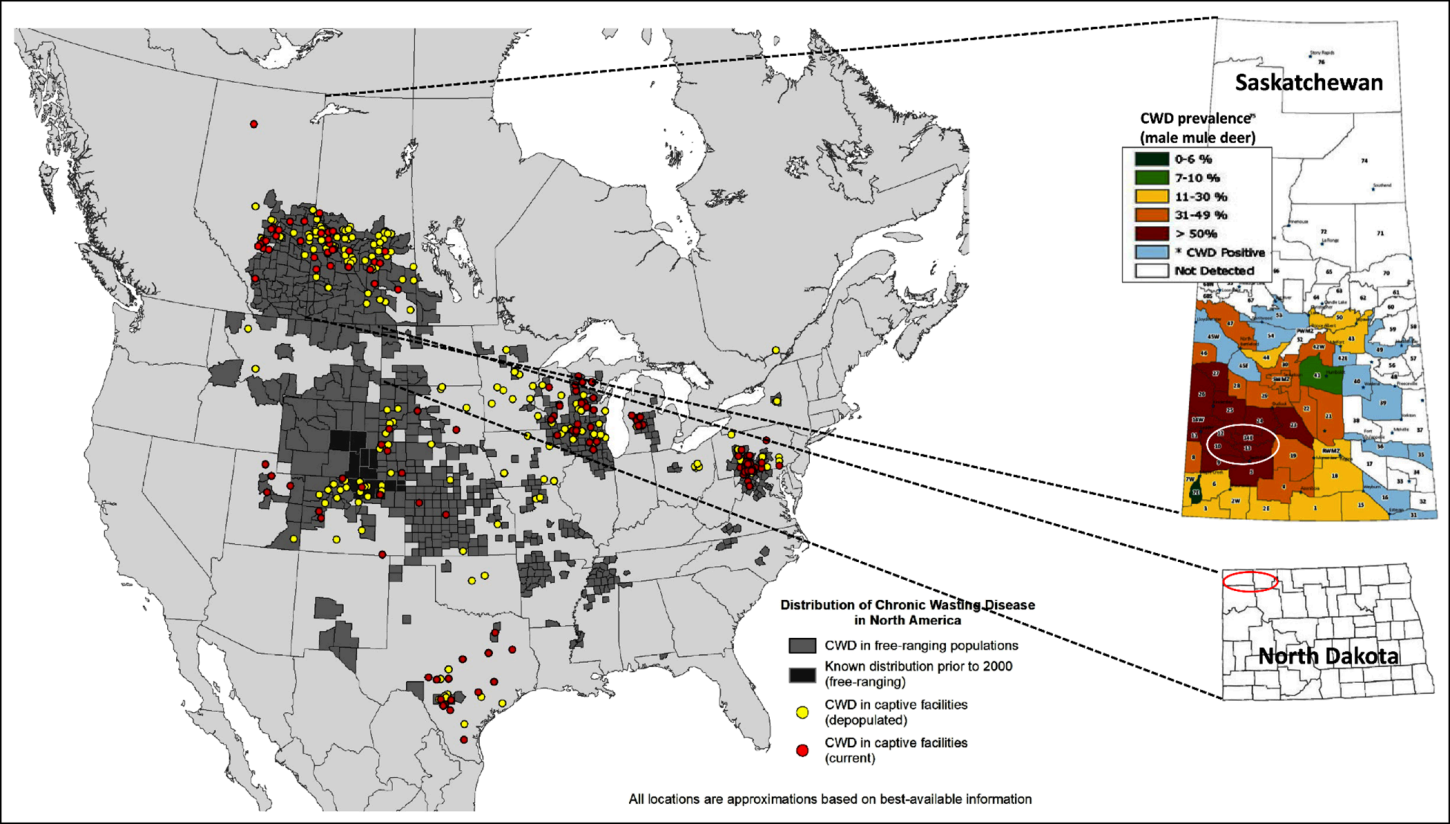
Soil sample collection areas (red and white ovals) in North Dakota (Divide County) and Saskatchewan (South Saskatchewan River valley). Soil samples were collected from the top 5 cm of A/Ah horizon; 5 subsamples from each site (1m apart). “Distribution of CWD in North America” map source: USGS National Wildlife Health Center; public domain. Insert “CWD prevalence in SK” map source: Government of Saskatchewan; public domain.

CWD was first detected in Saskatchewan farmed cervids in 1996 and in wild cervids in 2000, and it has since spread to other areas of Saskatchewan. It is considered endemic across the grassland and parkland regions of Saskatchewan, with the highest prevalence in the southwest region (Figure 3). In Saskatchewan, based upon previous research on mule deer behavior, movement and CWD prevalence in the province, an area of high CWD prevalence within, the SSRV, was selected for soil sampling.^34,35^ In North Dakota, the northwestern part of Divide County (bordering Saskatchewan) was chosen for soil sampling. In total, 25 sites in SK were sampled, and 13 sites were in ND (Table S1). We obtained 5 soil subsamples from the A/Ah horizons (0–5 cm) at each site, using a grid pattern. Even on contaminated sites, shed CWD prions do not distribute evenly (homogeneously) on the soil surface. To overcome this obstacle, from each site, we collected 5 soil subsamples/replicates spaced 1 m apart. Areas of interest included routes of group winter migration or those that were more localized, such as deer mortality sites and areas of historical grain spills and salt licks that attracted deer.^34^ Common deer habitats in southern SK, coulees, small and big ravines, were checked for recent deer activity, and soils were collected at different landscape positions: top, slope, or bottom of coulees. Areas with visual signs of deer presence, such as deer bed, feces, and fresh tracks, were sampled separately. Nearby areas with no visual recent deer activity (e.g., harvested field) were also sampled. We collected samples in the late stages of the vegetation season, specifically in September 2021 and August 2022. This occurred during a prolonged period of dry weather, with at least a week passing without any precipitation in both collection periods.

Soil subsamples were analyzed separately with 3 laboratory replicates of each subsample. Initially, we selected soils from the sites where we suspected the highest level of PrP^CWD^ (S1–S5) due to recent deer activity based on field observations. We attempted to detect PrP^CWD^ in these soils directly by immunoblotting without amplification. One gram of each soil was incubated with SB and then we analyzed supernatant using Western blot—PrP^CWD^ was not detected in any of these soils (Figure S3A). We tested one soil subsample from all 15 sites collected in 2021: soils were treated with SB and supernatants used as seeds for duplicate reaction in sPMCA. After 4 rounds of sPMCA, in soils S1, S2, S3, and S5, strong PrP^CWD^ amplification was observed in at least for one replicate with intensities ranging from 10 to 40% of the control (Figure S3B). Signals in soils S7, S8, S9, S12, S13, and S14 were weaker, with intensities less than 10%, but still stronger than the negative control (Figure S3C,D). Electrophoretic mobility of sPMCA products from field isolates often differed from that of the sPMCA products of positive controls. As noted by Saunders et al., the differences in sPMCA product sizes are likely due to loss of the N-terminus during desorption during the soil extraction process (Saunders et al., 2011).^13,24^

**Figure 4:**
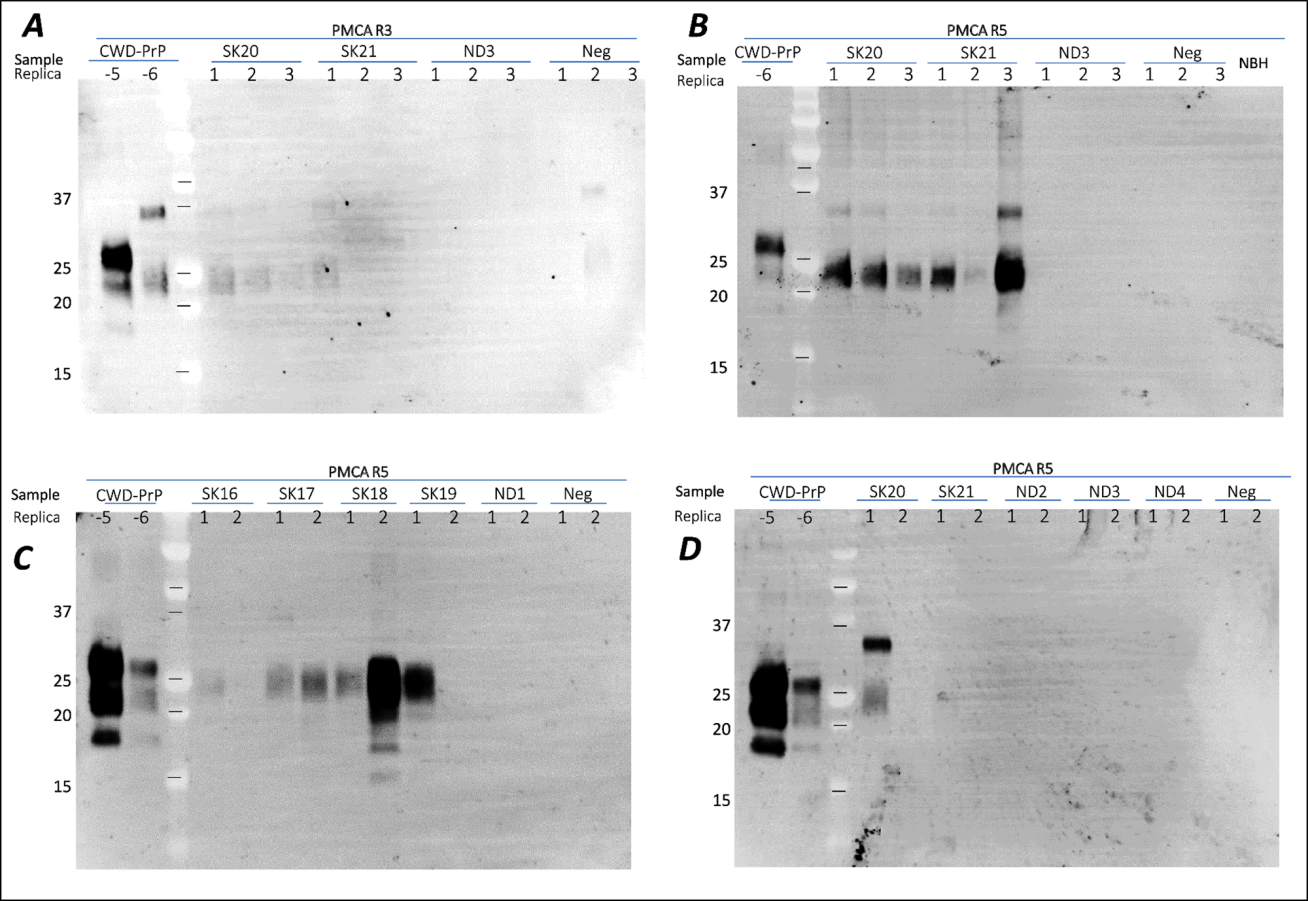
PrP^CWD^ detection in prairie soils collected in SK (S20 and S21) and ND (ND3) in 2022. Soil with similar properties from the CWD free region (Neg) was used as a control. PrP was extracted from soils with SB (1:1 ratio), and 10 μL of supernatant was used as a seed (in triplicate for panels A and B or duplicates for panels C and D) for sPMCA. PMCA products after 3rd (panel A) and 5th round (panels B–D) were PK-digested (75 μg/μL) and immunoblotted with mAb Bar224 (1:10,000).

**Figure 5:**
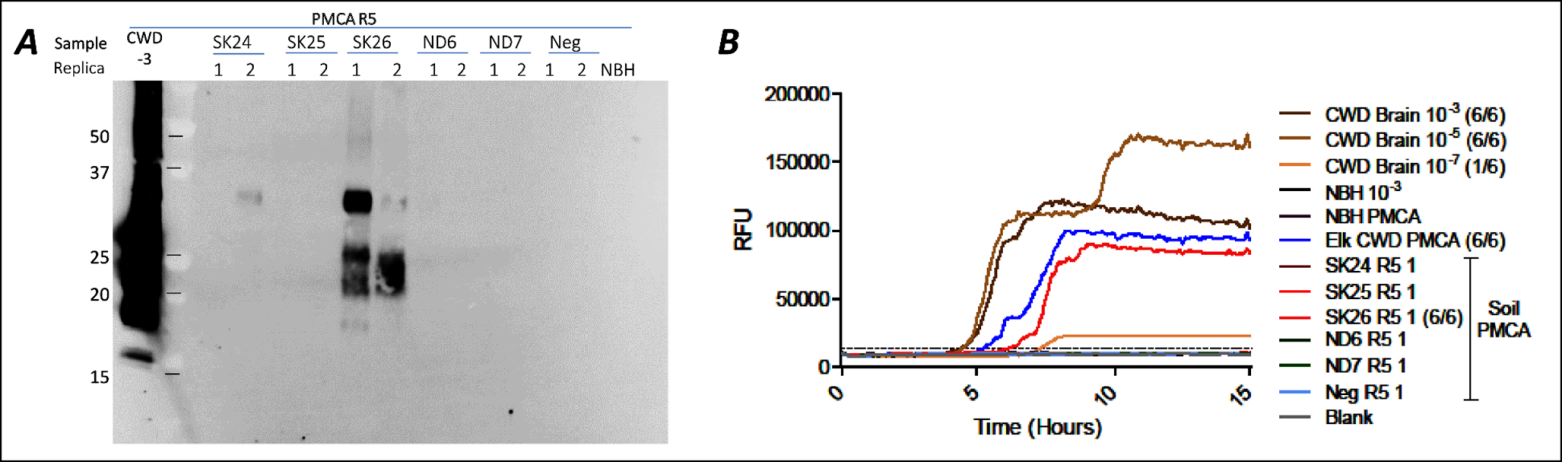
PrP^CWD^ detection in prairie soils collected in SK (S24, S25, and S26) and ND (ND6 and ND7) in 2022. Soil with similar properties (Neg) from the CWD free region was used as a control. PrP was extracted from soils with SDS-buffer (1:1 ratio), and 10 μL of supernatant was used as a seed (in duplicate) for sPMCA (Panels A). PMCA products (after round 5) were used for RT-QuIC reactions. The negative soil control and North Dakota sites did not produce a detectable RT-QuIC reaction, whereas CWD-positive controls and SK26 sample were positive by RT-QuIC (Panel B).

Specific soil samples collected from the SSRV, and adjacent coulees did not amplify detectable PrP^res^ in rounds 1 and 2 (data not presented). The samples exhibited PrP^res^ signals after conducting a minimum of 3 rounds of PMCA (Figure 4A), which intensified after a total of five rounds of PMCA (Figure 4B) and was amplified by RT-QuIC (Figure 5). Previously, for SSRV drainage basin, the surveillance model showed that the highest prevalence of CWD-positive deer was near small stream drainages.^36^ Most soils collected in SK demonstrated robust amplification after five rounds, whereas none of the soils from ND demonstrated amplification and subsequent seeding activity (Figure 4, Table 1).

**Table 1:** PrP^CWD^ Detected in Soils by sPMCA Followed by the RT-QuIC Method^a^

			**soils collected in 2021**		**soils collected in 2022**	
**location**	**soil type**	**deer activity**	**ID**	**PrP**^**CWD**^	**ID**	**PrP**^**CWD**^
**Saskatchewan**						
top of coulee	Orthic Regosol	no recent activity	S1	**+**	SK18	**+**
middle of coulee	Calcareous Regosols	positive deer carcass	S2	**+**	SK19	**+**
middle of coulee	Calcareous Regosols	fresh deer feces	S3	**+**	SK14	**+**
middle of coulee	Calcareous Regosols	old bones	S4	**+**	SK15	**+**
bottom of coulee	alluvium soil (weakly developed)	fresh deer bed	S5	**+**	SK16	**+**
harvested field	Brown Chernozem	no recent activity	S6	**–**	SK31	**–**
bottom of coulee	poorly drained alluvium soils	no recent activity	S7	**+**	SK28	**+**
abandoned old salt lick	Brown Chernozem	no recent activity	S8	**+**	SK27	**–**
farm yard, grain spill	Vertic Brown Chernozem	deer trail	S9	**–**		NA
farm yard, grain spill	Vertic Brown Chernozemic	no recent activity	S10	**–**		NA
grain bin near farm yard	Vertic Brown Chernozemic	no recent activity	S11	**–**		NA
soil under old feces	Orthic Brown Chernozem	old deer feces	S12	**+**		NA
soil under feces	Orthic Brown Chernozemic	deer trail	S13	**+**		NA
grain bin near farm yard	Vertic Brown Chernozemic	no recent activity	S14	**+**		NA
grain spill near grain bin	Vertic Brown Chernozemic	no recent activity	S15	**–**		NA
soil on coulee top	Calcareous Regosol	no recent activity		NA	SK17	**–**
soil on mid coulee	Calcareous Regosol	no recent activity		NA	SK20	**+**
soil on bottom	Orthic Regosols	fresh deer bed		NA	SK21	**+**
soil on bottom 2	Orthic Regosols	deer trail		NA	SK22	**+**
soil on coulee bottom	alluvium soil (weakly developed)	no recent activity		NA	SK23	**–**
soil on coulee top	Vertic Brown Chernozemic	no recent activity		NA	SK24	**–**
soil on coulee farm yard top	Vertic Brown Chernozemic	no recent activity		NA	SK25	**–**
soil on coulee bottom	poorly drained alluvium soils	fresh deer bed		NA	SK26	**+**
soil on trail in bush	poorly drained alluvium soils	deer trail		NA	SK29	**+**
soil near salt lick fresh	Vertic Brown Chernozemic	no recent activity		NA	SK30	**+**
**North Dakota**						
middle of coulee	Dark Brown Chernozem	no recent activity		NA	ND1	**–**
bushes between fields	Dark Brown Chernozems			NA	ND2	**–**
field	Dark Brown Chernozem (vertic)			NA	ND3	**–**
lowland near water body	Calcareous Regosol			NA	ND4	**–**
big ravine bottom	Dark Brown Chernozems			NA	ND5	**–**
middle of big ravine	Orthic Kashtanozems			NA	ND6	**–**
top of big ravine	Dark Brown Chernozem			NA	ND7	**–**
ravine between fields, bottom	Dark Brown Chernozem			NA	ND8	**–**
ravine between fields, middle	Orthic Kashtanozems			NA	ND9	**–**
field (wheat)	Dark Brown Chernozem			NA	ND10	**–**
ravine between fields, bottom	Orthic Kashtanozems			NA	ND11	**–**
side of the road	Orthic Regosol			NA	ND12	**–**
field (peas)	Dark Brown Kashtanozems			NA	ND13	**–**

a Soils were collected from sites with recent and past deer activities in South Saskatchewan and North Dakota. Positive detection of PrP^CWD^ (+) means that at least one replicate of the PMCA product after sPMCA has seeding activity on RT-QuIC. NA—not analyzed because soil samples were not collected from this site in 2021 or 2022.

The PMCA products (after 5 rounds) were tested for the presence of misfolded PrP using RT-QuIC from positive and negative sites (Table 1 and Table S3). The positive Western blot sample SK26 (Figure 5A) showed strong amplification on RT-QuIC, comparable to the PMCA amplification control tgElk BH. All negative samples (SK24, SK25, ND6, and ND7) tested negative in both PMCA and RT-QuIC (Figure 5B). One ND site (ND8) was negative on Western blot, but 2/6 replicates for one PMCA product crossed the positive threshold for RT-QuIC. The site was considered negative, but the weak RT-QuIC amplification suggests a very low PrP^CWD^ presence at the site. For sites where at least one of the soil subsamples was positive (S1, S2, S3, S4, and S5) by PMCA (Table S3), RT-QuIC showed amplification for all replicates for these soils. The addition of RT-QuIC to sPMCA provided a quantitative readout to the qualitative evaluation of sPMCA immunoblotting.

Of the 31 soils analyzed from SK and from the 13 from ND, 8 SK soils and all ND soils tested negative (Table 1 and Table S2). Notably, ND soils showed positive amplification after spiking with CWD-positive BH (Figure S4), indicating the ND soils do not inhibit PMCA reactions. Positive soil samples correlated with evidence of past and recent deer activity. The negative SK soils were collected in close proximity to positive soils, yet they were taken from an area devoid of any observable deer activity. In the positive SK soils, not all replicates and subsamples tested positive, with a variability in positivity rates ranging from 30 to 100% (Table S2). Interestingly, soil sites sampled in 2 consecutive years showed similar results: soils sampled in 2021 and identified positive showed positivity again when we collected them in 2022 from the same sites (i.e., S1 and SK18, S2 and SK19, S3 and SK14, S4 and SK15, etc.).

Reasons for such consistency may include (i) PrP^CWD^ persisting in the environment for more than a year and/or (ii) repeated deposition of PrP^CWD^ in the same area for two consecutive years. The first explanation may be supported by the detection of PrP^CWD^ at sites with and without recent deer activity. This would underpin the notion that detectable PrP^CWD^ remains in soil for extended periods following deposition from CWD-infected deer. Nevertheless, further research is needed to assess the long-term persistence of naturally shed CWD prions in the environment.

Analyses of different soil subsamples from one site (collected 1m apart) showed heterogeneity of PrP^CWD^ distribution on the landscape. For example, in one soil subsample, seeding activity was high and PrP^CWD^ amplified in all replicates, but in another soil subsample collected from the same site, no PrP^CWD^ was detectable after 5 rounds of PMCA and 20 h of RT-QuIC reaction (Table S2). For example, among soil subsamples collected from site S12 in 2021, subsamples A and C were positive and subsample B was negative. If at least one soil subsample from the site was positive, we defined this site as positive for PrP^CWD^ (Table 1).

## Implications

This study demonstrates that, in a variety of soils of varying mineralogy and texture, PrP^CWD^ can be detected in environmental soil samples in areas of high disease prevalence using SB extraction followed by the sPMCA and RT-QuIC. This represents a significant improvement in soil-bound PrP^CWD^ detection, benefiting both surveillance and mitigation approaches. Not unexpectedly, some soil subsamples, collected in close proximity to positive subsamples, were devoid of detectable PrP^CWD^ seeding activity, suggesting that CWD infectivity is not homogeneously distributed in a landscape with high prevalence of CWD. Therefore, a negative result for a series of soil samples would not be a proof of a complete absence of CWD prions in soil because of uneven prion distribution on the soil surface.

Repeated detection of PrP^CWD^ in soils collected from the same sites in two consecutive years suggests persistence of CWD in the environment. Understanding the distribution of contaminated soils in the landscape may help prioritize intervention strategies to control CWD spread in deer populations. CWD management strategies targeting the decontamination of CWD-affected soils or restricting access for deer to these areas may be useful tools for future consideration. Several future directions to improve our understanding of the persistence and spread of PrP^CWD^ in soil are suggested, including optimization of detection methods for other soil types, quantifying infectivity levels (linking detection with infectious dose), and investigation of seasonal dynamics and soil variability impacts on prion persistence. Overall, these findings have important implications for the management and prevention of CWD in both wild and captive cervid populations. The detection of PrP^CWD^ in prairie soils in Saskatchewan with a high CWD prevalence highlights the potential for environmental contamination to play a role in CWD transmission.

## Supporting Information Available

(Table S1) Soils collected from sites with recent and past deer activity in South Saskatchewan and North Dakota at the end of vegetation seasons in 2021 and 2022, (Table S2) PrP^CWD^ detected in soils collected from sites with recent and past deer activity in South Saskatchewan and North Dakota, (Figure S1) PrP extracted from soil with different extractants, (Figure S2) PrP^CWD^ detection in prairie soils collected in SK in 2021, and (Figure S3) PrP^CWD^ detection in prairie soils collected in SK in 2022 (PDF)
