# Supplementary material for: Detection of Chronic Wasting Disease Prions in Prairie Soils from Endemic Regions
Source: Environ Sci Technol. 2024 Jun 12;58(25):10932–40. doi: 10.1021/acs.est.4c04633 (PMC11210205; doi:10.1021/acs.est.4c04633)
Supplement: Supplementary file 1 — es4c04633_si_001.pdf [file es4c04633_si_001.pdf]

Supporting information

Detection of chronic wasting disease prions in prairie soils from endemic regions

Alsu Kuznetsova[a,b], Anthony Ness[b], Erin Moffatt[c], Trent Bollinger[c], Debbie McKenzie[b], Iga Stasiak[d], Charlie S. Bahnson[e], Judd M. Aiken[b*]

[a] *Department of Renewable Resources, University of Alberta, Edmonton, T6G 2G7, Canada*

[b] *Centre for Prions and Protein Folding Diseases, University of Alberta, Edmonton, T6G 2M8, Canada*

[c] *Canadian Wildlife Health Cooperative Western Northern, University of Saskatchewan, Saskatoon, S7N 5B4, Canada*

[d] *Ministry of Environment, Government of Saskatchewan, Saskatoon, S7N 3R3, Canada*

[e] *North Dakota Game and Fish Department, Bismarck, ND, 58501-5095, USA*

**Corresponding author: judd.aiken@ualberta.ca*

Summary: 10 pages, 2 tables, 4 figures.

Table S1. Soils collected from sites with recent and past deer activity in South Saskatchewan and North Dakota at the end of vegetation seasons in 2021 and 2022

Soils collected in 2021	Soils collected in 2022	Location	Soil types	Surface texture	Deer activity
S1	SK18	Top of coulee	A complex of soils formed in various deposits associated with steep and eroding valley sides: mainly orthic Regosols, with calcareous Regosols on upper slopes and knolls	Range from silt to heavy clay	No visual recent activity, 20m away from carcass
S2	SK19	Middle of coulee			Carcass positive 1yo
S3	SK14	Middle of coulee			Feces 2-5m away
S4	SK15	Middle of coulee			Bones
S5	SK16	Bottom of coulee	Mainly a mixture of weakly developed Alluvium soils formed in variable-textured alluvial materials	Clay	Deer bed, deer trail
S6	SK31	Harvested field	Brown Chernozemic soils formed in shallow, silty lacustrine materials underlain by glacial till	Silty clay	Harvested field, no visual recent activity
S7	SK28	Bottom of coulee	Mainly a mixture of weakly developed, poorly drained Alluvium soils on lower slopes and in depressions	Clay	No visual recent activity
S8	SK27	Abandoned old salt lick	Brown Chernozemic soils formed in shallow, silty lacustrine materials underlain by glacial till	Silty loam	Old salt lick, no visual recent activity (unidentified)
S9	NA*	Farm yard, grain spill	Vertic Brown Chernozemic soils formed in clayey lacustrine materials	Heavy clay	Deer trail
S10	NA	Farm yard, grain spill			No visual recent activity
S11	NA	Grain bin near farm yard			No visual recent activity
S12	NA	Soil under old feces	Mainly orthic Brown Chernozemic soils formed in variable, clayey lacustrine materials, with weakly developed orthic Brown Chernozemic soils intermixed	Silty clay	No visual recent activity
S13	NA	Soil under feces			Deer trail
S14	NA	Grain bin near farm yard	Grumic Brown Chernozemic soils formed in clayey lacustrine materials	Heavy clay	No visual recent activity
S15	NA	Grain spill near grain bin			No visual recent activity
NA	SK17	Soil on coulee top	Complexes of orthic Regosols with calcareous Regosols	Range from silt to heavy clay	No visual recent activity
NA	SK20	Soil on mid coulee			No visual recent activity
NA	SK21	Soil on bottom, under mint bed	Mainly a mixture of weakly developed, poorly drained Alluvium soils on lower slopes and in depressions	Clay loams	Deer bed
NA	SK22	Soil on bottom 2			Deer trail
NA	SK23	Soil on coulee bottom watering	Weakly developed Alluvium soils formed in variable-textured alluvial materials		No visual recent activity
NA	SK24	Soil on coulee top	Grumic Brown Chernozemic soils formed in clayey lacustrine materials	Loams	No visual recent activity
NA	SK25	Soil on coulee farm yard top			No visual recent activity
NA	SK26	Soil on coulee farm yard bottom under bed	Mainly a mixture of weakly developed, poorly drained Alluvium soils on lower slopes and in depressions	Clay	Deer bed
NA	SK29	Soil on trail in bush	Weakly developed Alluvium soils formed in variable-textured alluvial materials		Deer trail
NA	SK30	Soil near salt lick fresh	Grumic Brown Chernozemic soils formed in clayey lacustrine materials	Heavy clay	Salt lick with deer tracks
NA	ND1	Middle of coulee	Dark Brown Chernozems associated with calcareous Regosols and Solonetz (Williams series)	Loams to clay loams	No visual recent activity
NA	ND2	Bushes between fields			Deer feces and trail
NA	ND3	Field			No visual recent activity

NA	ND4	Lowland near water body			No visual recent activity
NA	ND5	Big ravine bottom	Complexes of Dark Brown Chernozems with Kashtanozems (Chestnut soil, Williams series)		No visual recent activity
NA	ND6	Middle of big ravine			No visual recent activity
NA	ND7	Top of big ravine			No visual recent activity
NA	ND8	Ravine between fields, bottom			No visual recent activity
NA	ND9	Ravine between fields, middle			No visual recent activity
NA	ND10	Field			No visual recent activity
NA	ND11	Ravine between fields, bottom			Deer bed
NA	ND12	Side of the road	Orthic Regosols (Williams series)	Loams	No visual recent activity
NA	ND13	Field	Dark Brown Kashtanozems (Williams series)	Clay loams	No visual recent activity

*Soil samples were not collected from this site

Table S2. Properties of collected soil samples

ID	Soil	Location	Horizon	pH	TOC, %	HA content, g L^{-1}	Texture	Mineralogy of clay fraction
**Soils used for spiked experiments**								
Soil 1	Orthic Dark Brown Chernozem	South-central region, Alberta, Canada	Ah	7.9	3.9	15.1	Clay loam	Mte-Kte
Soil 2	Gleyed Dystric Brunisol	Mountain region, Old Entrance, Alberta, Canada	Bf	6.3	1.0	0.5	Loam	Mica-illite
**Soils collected in SK and ND**								
S1/SK18	Orthic Regosol	Top of coulee, SSRV, SK, Canada	A	6.3	0.8	8.0	Silty clay	Mte-Kte
S5/SK16	Alluvium soil (weakly developed)	Bottom of coulee, SSRV, SK, Canada	A	6.8	0.7	8.2	Clay	NA*
SK31	Brown Chernozem	Harvested field, SSRV, SK, Canada	Ap	6.8	2.5	12.0	Silty clay	Mte-Kte
S8/SK27	Brown Chernozem	Abandoned old salt lick, SSRV, SK, Canada	Ah	6.9	2.1	14.5	Silty loam	NA
S9	Vertic Brown Chernozem	Farm yard, grain spill, SSRV, SK, Canada	Ah	6.5	3.2	21.8	Heavy clay	NA
S12	Orthic Brown Chernozem	Grain bin near farm yard, SSRV, SK, Canada	Ah	7.5	1.8	12.5	Silty clay	Mte-Kte
SK17	Calcareous Regosol	Soil on coulee top, SSRV, SK, Canada	A	7.6	0.9	6.5	Silty clay	NA
SK23	Alluvium soil (weakly developed)	Soil on coulee bottom watering, SSRV, SK, Canada	Ah	7.3	2.6	9.1	Clay loams	NA
ND1	Dark Brown Chernozem	Middle of coulee, ND, USA	Ah	7.5	1.8	19.2	Clay loams	Mte-Kte
ND3	Dark Brown Chernozem	Field, ND, USA	Ap	7.8	2.5	18.4	Silty loam	Mte-Kte
ND4	Calcareous Regosol	Lowland near water body, ND, USA	Ah	7.9	2.9	11.2	Clay loams	NA
ND7	Dark Brown Chernozem	Top of big ravine, ND, USA	Ah	6.9	2.5	15.9	Silty loam	NA
ND8	Dark Brown Chernozem	Ravine between fields, bottom, SSRV, ND, USA	Ah	7.1	2.9	22.6	Clay loams	NA
ND12	Orthic Regosol	Side of the road, ND, USA	Ah	7.4	1.9	15.9	Loam	Mte-Kte

*NA – not analysed

Table S3. PrP^{CWD} detected in soils collected from sites with recent and past deer activity in South Saskatchewan and North Dakota. Numbers in "PMCA results" and "RT-QuIC" columns represent positive replicates after sPMCA or RT-QuIC reactions, respectively. For example, 2/2 means 2 positive replicates were detected out of 2 total replicates.

**Location**	Soils collected in 2021				Soils collected in 2022			
	ID	**PrP^{CWD}**	**PMCA**	**RT-QuIC**	ID	**PrP^{CWD}**	**PMCA**	**RT-QuIC**
				**Saskatchewan**				
Top of coulee	S1	+	2/2; 2/2; 2/2; 2/2; 0/2	6/6; 4/6	SK18	+	2/2	6/6; 6/6
Middle of coulee	S2	+	1/2; 2/2; 2/2; 1/2; 2/2	6/6; 6/6	SK19	+	1/2; 2/3; 2/2	6/6; 0/6
Middle of coulee	S3	+	2/2; 2/2; 1/2; 2/2; 2/2	6/6; 6/6	SK14	+	2/3; 3/3; 2/2	6/6; 6/6
Middle of coulee	S4	+	0/2; 2/2; 0/2; 1/2	6/6; 5/6	SK15	+	1/3; 3/3	6/6; 6/6
Bottom of coulee	S5	+	1/2; 1/2; 1/2; 2/2; 0/2	5/6; 5/6	SK16	+	0/3; 3/3	6/6; 6/6
Harvested field	S6	-	0/2; 0/2; 0/2	0/6; 0/6	SK31	-	0/3; 0/2	0/6; 0/6
Bottom of coulee	S7	+	0/2; 2/2; 1/2	6/6; 6/6	SK28	+	0/2	6/6; 0/6
Abandoned old salt lick	S8	+	0/2; 1/2; 1/2	6/6; 3/6	SK27	-	1/2	0/6
Farm yard, grain spill	S9	-	1/2; 1/2; 2/2	0/6; 0/6	NA			
Farm yard, grain spill	S10	-	0/2; 0/2; 0/2	0/6; 0/6	NA			
Grain bin near farm yard	S11	-	0/2; 0/2	0/6; 0/6	NA			
Soil under old feces	S12	+	2/2; 0/2; 2/2	6/6; 6/6	NA			
Soil under feces	S13	+	0/2; 0/2; 1/2	6/6; 0/6	NA			
Grain bin near farm yard	S14	+	2/2; 0/2; 2/2	6/6; 6/6	NA			
Grain spill near grain bin	S15	-	2/2; 0/2; 0/2	0/6; 0/6	NA			
Soil on coulee top	NA				SK17	-	0/3; 0/3	0/6; 0/6
Soil on mid coulee	NA				SK20	+	2/2; 2/3	6/6; 0/6
Soil on bottom, under mint bed	NA				SK21	+	2/2; 1/3	0/6; 0/6
Soil on bottom 2	NA				SK22	+	1/2; 2/3	0/6; 6/6
Soil on coulee bottom watering	NA				SK23	-	1/2; 0/3	0/6; 0/6
Soil on coulee top	NA				SK24	-	0/2	0/6
Soil on coulee farm yard top	NA				SK25	-	0/2	0/6

Soil on coulee farm yard bottom under bed	NA		SK26	**+**	2/2	6/6
Soil on trail in bush	NA		SK29	**+**	1/2	6/6; 0/6
Soil near salt lick fresh	NA		SK30	**+**	1/2	6/6; 6/6
**North Dakota**						
Middle of coulee	NA		ND1	**-**	0/2; 0/2	0/6; 0/6
Bushes between fields	NA		ND2	**-**	0/2	0/6; 0/6
Field	NA		ND3	**-**	0/3; 0/2	0/6; 0/6
Lowland near water body	NA		ND4	**-**	0/2	0/6; 0/6
Big ravine bottom	NA		ND5	**-**	0/2	0/6; 0/6
Middle of big ravine	NA		ND6	**-**	0/2	0/6; 0/6
Top of big ravine	NA		ND7	**-**	0/2	0/6; 0/6
Ravine between fields, bottom	NA		ND8	**-**	0/2	0/6; 2/6
Ravine between fields, middle	NA		ND9	**-**	0/2	0/6; 0/6
Field	NA		ND10	**-**	0/2	0/6; 0/6
Ravine between fields, bottom	NA		ND11	**-**	0/2	0/6; 0/6
Side of the road	NA		ND12	**-**	0/2	0/6; 0/6
Field	NA		ND13	**-**	0/2	

NA – not analysed because soil samples were not collected from this site

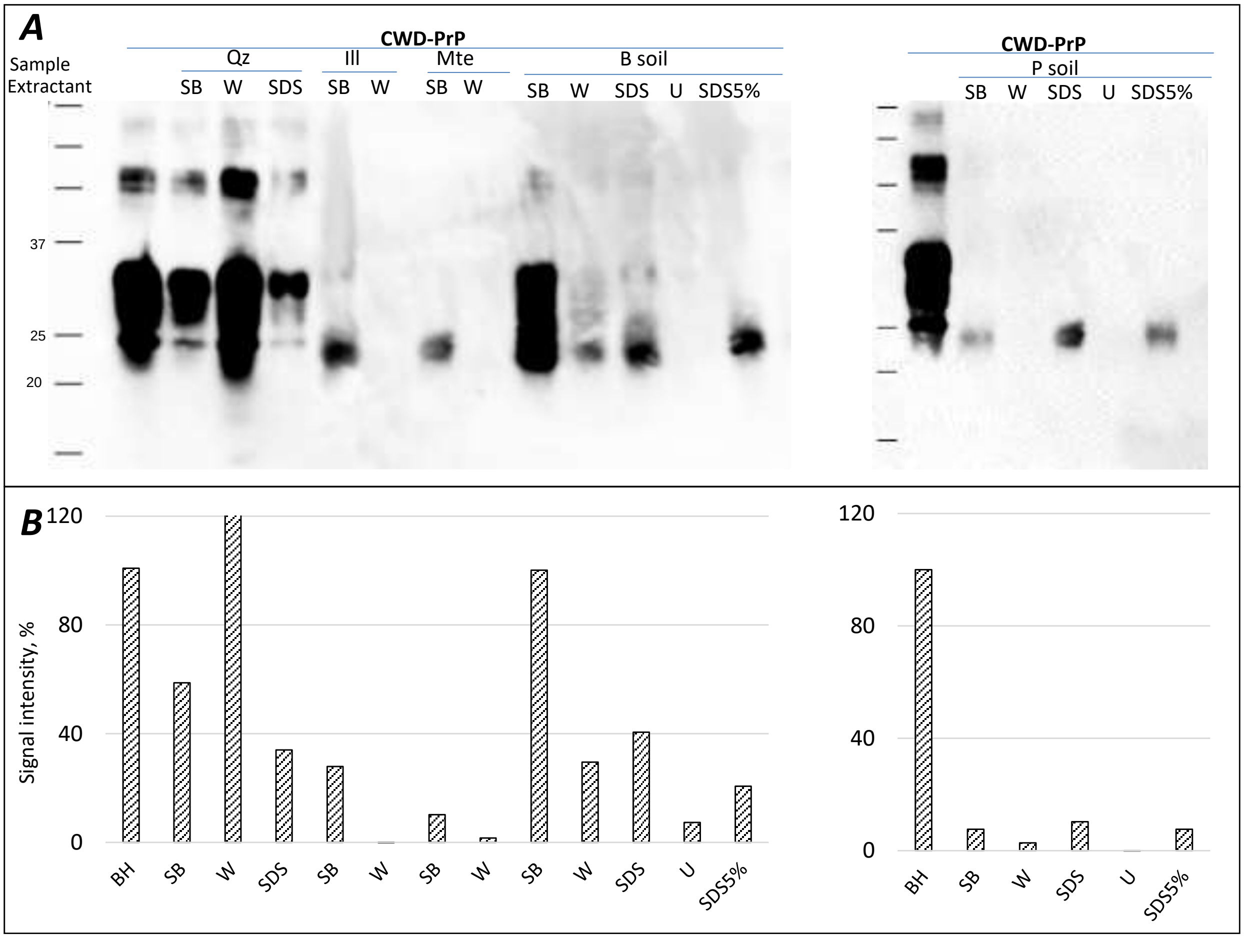

Figure S1. PrP extracted from soil with different extractants. Panel A: Highest PrP recovery from soils and minerals was attained with sample buffer 5xLaemmlii (SB) and SDS 10%. 100 mg of soils (B soil – Boreal soil, Luvisol and P soil – Prairie soil, Chernozem) and minerals (Qz – quartz, Ill – illite, Mte – montmorillonite) were spiked with 10 µL of 1% tgElk-CWD BH and incubated at 4˚C at the dark for 1 day. PrP was extracted with SB, W – deionised water, SDS - SDS 5 or 10%, or U – urea 2% at 80˚C for 10 min and immunoblotted with mAb Bar224 (1:10,000). Panel B: quantification of PrP signal using ImageJ software. Initial amount of BH was considered as 100% of signal intensity.

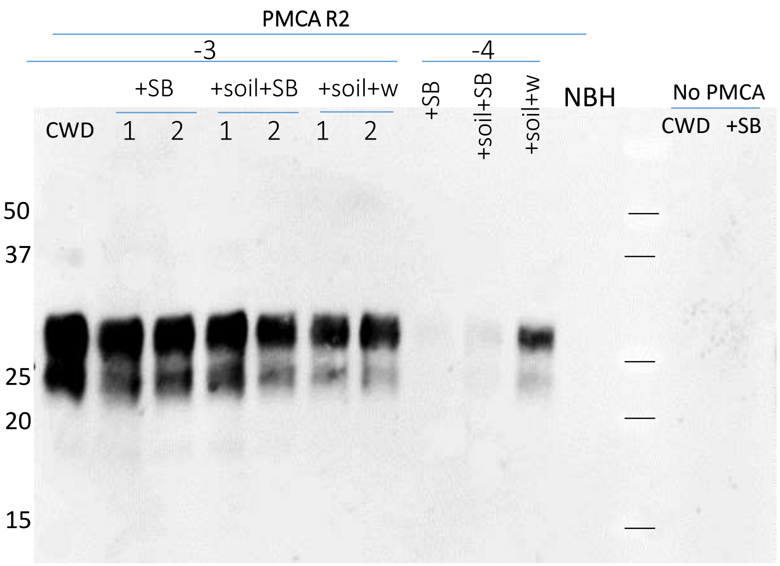

Figure S2. Extraction buffer and soil addition does not affect PrP^{CWD} detection in spiked soils using PMCA: 10µL of 1% tgElk-CWD BH was mixed with SB, or soil+SB, or soil+water. Supernatant (10µL) from these samples were used as a seed for PMCA (samples with -3 dilution) in duplicate; for -4 dilution, these samples were diluted further. CWD-BH in dilution 10^{-3} (first line) and uninfected BH (NBH) were used as a PMCA amplification controls. PMCA products were PK-digested (50 µg/µL) and immunoblotted with mAb Bar224.

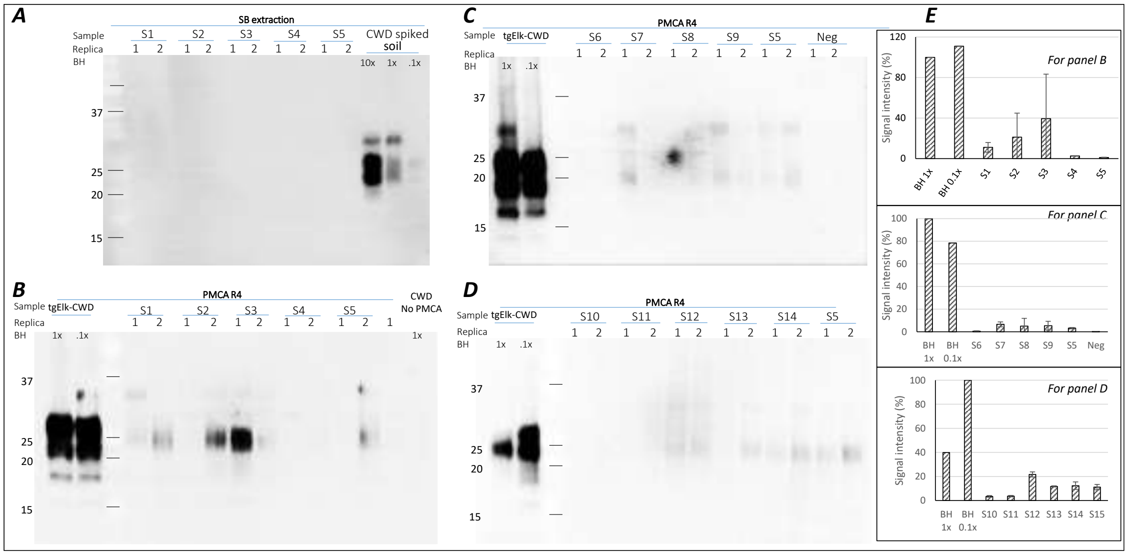

Figure S3. PrP^{CWD} detection in prairie soils collected in SK in 2021. Panel A: PrP was extracted from 5 soils in duplicate and analyzed by immunoblotting. As a control, one soil (S1) was spiked with 10^{-2} -10^{-4} PrP^{CWD} and then extracted PrP^{CWD} similarly to non-spiked soils. Last 3 lines (Panel A) show efficiency of the SB extraction from this soil after spiking with PrP^{CWD}. Panels B, C and D: PrP was extracted from soils with SB (1:1 ratio) and 10µL of supernatant was used as a seed for sPMCA. PMCA products (after 4th round) were PK-digested (50 µg/µL) and immunoblotted with mAb Sha31. Panels A and B: soils from the sites with recent deer activity; panels C and D: soils from the sites with past (>2 years) deer activity. Soil from CWD-free region (Neg) was used as a negative control. Panel E: quantification of PrP signal using ImageJ software. Initial amount of BH was considered as 100% of signal intensity.

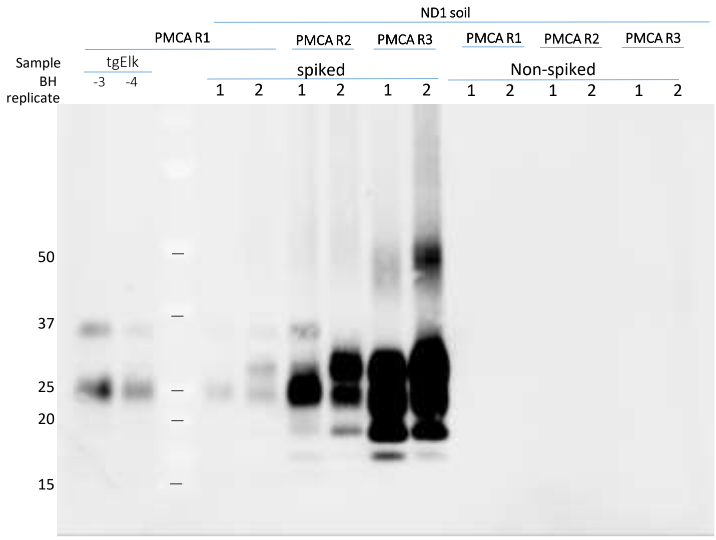

Figure S4. PrP^{CWD} detection in spiked soil collected in ND using PMCA. Soil (100 mg) were spiked with 10 µL of 1% tgElk-CWD BH, incubated at 4ºC at the dark for 1 day. PrP was extracted from soils with SB and 10µL of supernatant was used as a seed for PMCA. Same soil was treated with SB (without spiking) and also used as a seed for PMCA. CWD-BH in dilution 10^{-3} - 10^{-4} and uninfected BH (NBH) were used as a PMCA amplification controls. PMCA products (after rounds 1, 2 and 3) were PK-digested (50 µg/µL) and immunoblotted with mAb Bar224.
